# N-Losses and Energy Use in a Scenario for Conversion to Organic Farming

**DOI:** 10.1100/tsw.2001.305

**Published:** 2001-11-16

**Authors:** Tommy Dalgaard, Chris Kjeldsen, Nicholas J. Hutchings, Jorgen F. Hansen

**Affiliations:** Danish Institute of Agricultural Sciences, Department of Agricultural Systems, Tjele, Denmark

**Keywords:** nitrogen, fossil energy, organic farming, Denmark, nitrogen, scenario, crop-rotation, fertilisation

## Abstract

The aims of organic farming include the recycling of nutrients and organic matter and the minimisa-tion of the environmental impact of agriculture. Reduced nitrogen (N)-losses and energy (E)-use are therefore fundamental objectives of conversion to organic farming. However, the case is not straightforward, and different scenarios for conversion to organic farming might lead to reduced or increased N-losses and E-use. This paper presents a scenario tool that uses a Geographical Information System in association with models for crop rotations, fertilisation practices, N-losses, and E-uses. The scenario tool has been developed within the multidisciplinary research project Land Use and Landscape Development Illustrated with Scenarios (ARLAS). A pilot scenario was carried out, where predicted changes in N-losses and E-uses following conversion to organic farming in areas with special interests in clean groundwater were compared. The N-surplus and E-use were on average reduced by 10 and 54%, respectively. However, these reductions following the predicted changes in crop rotations, livestock densities, and fertilisation practices were not large enough to ensure a statistically significant reduction at the 95% level. We therefore recommend further research in how conversion to organic farming or other changes in the agricultural practice might help to reduce N-surpluses and E-uses. In that context, the presented scenario tool would be useful.

## INTRODUCTION

The agricultural use of fossil energy (E) and nitrogen (N) are important issues. On the one hand, the use of these resources is necessary to attain agricultural production, while on the other hand it leads to undesirable greenhouse gas emissions[1] and N-losses to the environment[2]. Knowledge about the advantages compared to the environmental and socioeconomic costs in the use of these and other resources is therefore needed and has been a focus area for scientific research. Conversion to organic farming might be a useful measure to reduce both N-losses[3] and E-use[4]. However, the case is not straightforward, and different scenarios for conversion to organic farming might lead to reduced or increased N-losses and energy consumption.

The present study aims to present a method to map, simulate, and compare N-losses and E-uses at the regional level. The method is demonstrated in a Danish study area. Especially, it is the aim to compare the present situation to a pilot scenario for conversion to 100% organic farming in those parts of the study area with special interests to protect the groundwater.

## MATERIALS AND METHODS

The study area was situated around the city of Bjerringbro in the midwest of Denmark and covered 10 × 10 km$^2$. A 1998 land-use map of this area was digitised in scale 1:10,000, based on a digital field-block map (Table 1), aerial photography, and satellite images combined with ground-truth observations (Fig. 1). The area included 65%

**TABLE 1**
**Data Collected in the 10- × 10-km² Study Area**

| | Type | Sources[a] |
|---|---|---|
| 1. | Livestock types and numbers for each farm | The Danish Livestock Register (CHR) |
| 2. | Crop type and area for each field | The Danish Field Crop Register (GLR) |
| 3. | Fertilisation practice for each farm | The Danish Register of Fertilisation Accounts |
| 4. | Link between land owner and land farmer | The Cadastral Register of Denmark (ESR) |
| 5. | Geographical placement of fields | The field-block map (DMK-Block) and aerial photography |
| 6. | Geographic placement of groundwater protection areas | The County Register over areas designated |

[a] Most of these data sources are included in the Ministry of Food, Agriculture, and Fisheries' national databases[5].

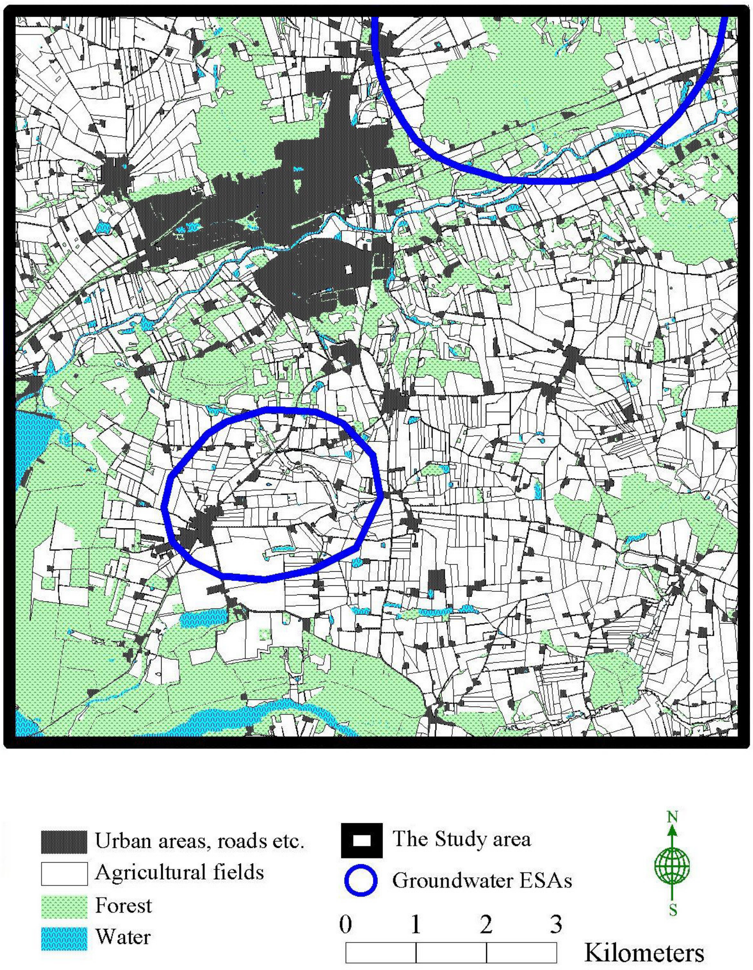

**FIGURE 1**. Land use in the 10- × 10-km² study area, situated around the city of Bjerringbro in the midwest of Denmark. This study is limited to describing the N-losses and the E-use related to the agricultural areas within or outside the two environmentally sensitive areas (ESAs) with special interests in clean groundwater.

agricultural land and 20% forests, and the remaining area consisted of urban areas, roads, lakes, streams, hedges, and other small biotopes. The present study was limited to N-losses and E-use related to the agricultural land. Of this agricultural land, 16% of the area was designated as environmentally sensitive areas (ESAs) with special interests to protect the groundwater.

Agricultural land-use data were collected from the Ministry of Food, Agriculture, and Fisheries' national databases (Table 1). This information was collected for all 489 farms within the region in 1998. Moreover, detailed information about farm-level N-inputs (i1–i7) and N-outputs (o1–o4) were collected from a sample covering 41 farms within the study area (Fig. 2). Thereby, the N-surpluses from these farms were obtained, and a nonlinear relation[6] between the N-surplus and the fertilisation in the form of animal manure (N-am) was derived (Fig. 3). Analogously, a function was derived for organic farming on the basis of simulated results from a previous study in the area[6] (Fig. 3). In this study, the N-surpluses were used to indicate the overall N-losses from agriculture in the form of dissolved N (mainly nitrate), gaseous N (ammonia, dinitrogen, nitrogen oxides, etc.), and solid N (mainly organic matter).

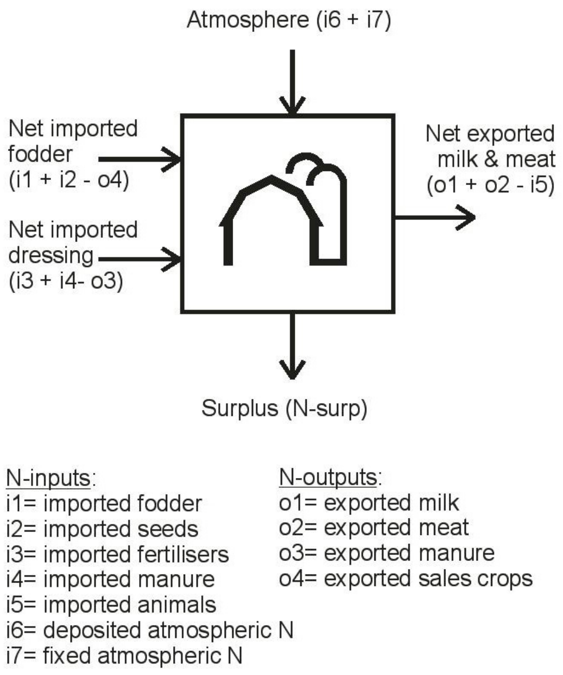

**FIGURE 2**. Farm N-inputs (i1–i7), N-outputs (o1–o4), and N-surplus, i.e., in kg N ha$^{-1}$ year$^{-1}$[3]. **[[fig taken from Ref[3]?]]**

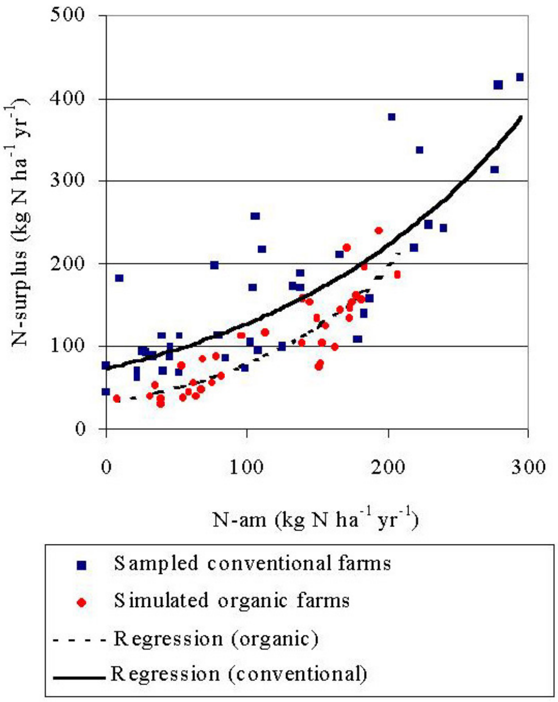

**FIGURE 3**. Relationship between nitrogen surplus (N-surplus) and the farm-level, ex-store application of nitrogen in the form of animal manure (N-am) in conventional farming and organic farming[6].

In a geographical information system, data on land use and ESAs with special interests in clean groundwater (Fig. 1, Table 1) were used to construct a scenario for conversion to organic farming. In this scenario, all farms with fields within the two ESAs were converted to organic farming. This implied a reduction in the livestock density. The herds on organic livestock farms where the livestock density exceeded 1.4 livestock units (LSU) per hectare, corresponding to the maximum allowed livestock density according to Danish organic-farmer regulations, were cut to that limit. Also the crop rotations, crop yields, fertilisation practices, required fodder imports, and types of field operations were affected by the conversion. The simulations are explained in detail in the following.

The collected data, in combination with information about 10 years of practice on organic farms in Denmark[7], were used to simulate crop rotations[8] and fertilisation practices[9] on organic and conventional farms. This simulation meant that the arable crop and corresponding fertilisation practice were decided for each field in 30 succeeding years, given the present management practice. The results presented are the averages of these 30-year periods, such as the fertiliser use presented in Table 2. For example, a typical crop rotation on conventional dairy farms would be fodder beets → spring barley → spring barley, undersown grass → grassland in rotation → 1st year grassland → 2nd year grassland → winter wheat → spring barley → spring barley and peas for silage, undersown grass → 1st year grassland, etc. The typical rotation on organic farms would consist of more grass/clover and would be simpler, for example, spring barley, undersown grass/clover → 1st year grass/clover → 2nd year grass/clover, etc. Moreover, some of the area would be out of rotation and covered by permanent grassland or permanent set-aside (Table 3). The level of fertilisation on conventional farms was simulated to correspond to the recorded, present fertilisation level (Table 1). Organic farms only used livestock manure. If the manure production on either an organic or a conventional farm exceeded the norms for fertilisation according to Danish legislation[9], the manure was transported to the nearest farm without manure surplus. Finally, the farm-level N-fertiliser amounts were distributed to the corresponding fields according to current practice[9]. For example, spring barley on loamy soils at conventional farms would be fertilised with 123 kg plant-available N ha$^{-1}$ year$^{-1}$, corresponding to a yield of 6 t cereals ha$^{-1}$ yr$^{-1}$. The same crop on organic farms would be fertilised with around 90 kg plant-available N ha$^{-1}$ year$^{-1}$, corresponding to a yield of 4 t cereals ha$^{-1}$ year$^{-1}$. The simulated yields corresponded to published crop yields and crop yield losses when converting to organic farming in Denmark[10].

The fossil E-use, measured in Joules (J), was simulated with Dalgaard et al.'s model[11]. This model divides the E-use into energy use for crop and livestock production. Moreover, the energy use is divided into direct and indirect E-use. Direct E-use is energy input used in the production, when such input can be directly converted into energy units (e.g., diesel fuel, lubricants, and electricity for lubrication and drying). Indirect

**TABLE 2**
**Farm-Level N-Surpluses (kg N ha$^{-1}$ year$^{-1}$), Before and After Conversion to Organic Farming, in the Environmentally Sensitive Areas (ESAs) with Special Interests in Clean Groundwater**

| | Within ESAs (n = 61)[a] | Outside ESAs (n = 428) | Total (n = 489) |
|---|---|---|---|
| Before conversion | 115 ± 15 | 107 ± 5 | 108 ± 5 |
| After conversion | 104 ± 14 | [b] | 107 ± 5 |

[a] n = number of farms.
[b] The farms outside ESAs were not converted and have the same nitrogen surpluses as before.

**TABLE 3**
**Field-Crops in the Study Area in the Present Situation (P) and in the Scenario (S) with Conversion to Organic Farming in the Environmentally Sensitive Areas (ESAs) with Special Interests in Clean Groundwater**

| | Within ESAs | | Outside ESAs | Total | |
|---|---|---|---|---|---|
| | P | S | P = S | P | S |
| Cereals | 60% | 53% | 66% | 65% | 64% |
| Row crops | 9% | 0% | 10% | 10% | 8% |
| Glass/clover in rotation | 5% | 24% | 4% | 4% | 7% |
| Perm. grassland or set-aside | 26% | 23% | 20% | 21% | 20% |

E-use is energy used in the production when such input cannot be converted directly into energy inputs (e.g., machinery, fertilisers, pesticides, and fodder imports). The needed conventional or organically produced fodder that was not produced within the 10- ×10-km$^2$ study area was supposed to be imported from outside the area. No manure was imported to the area, either in the present situation or in the scenario for conversion to organic farming.

The crops were divided into four types: cereals (spring cereals, winter cereals, and field-peas), row crops (beets, rape, and corn grown for fodder), grass/clover in rotation, and permanent grassland or set-aside grassland. The E-uses for each of these crop types were simulated from E-use standards for typical management practices in the form of field operations and use of input factors (nitrogen, phosphorous, potassium, lime, pesticides, machinery, and irrigation)[12]. Subsequently, the E-uses for these crop types were multiplied with the area of each crop type in the present situation and in the scenario for conversion to organic farming. Likewise, the livestock were divided into ruminants (cattle, sheep, goats, etc.) and nonruminants (pigs, poultry, etc.), and the average E-use was simulated per livestock unit of these two types, where one livestock unit corresponds to that number of animals producing 100 kg N ex store per year. Finally, the E-uses for crop and animal production were aggregated for each farm and used to calculate the average E-use before and after conversion (Table 4).

**TABLE 4**
**Farm-Level Energy Uses ($10^6$ J ha$^{-1}$ year$^{-1}$), Before and After Conversion to Organic Farming, in the Environmentally Sensitive Areas (ESAs) with Special Interests in Clean Groundwater**

| | Within ESAs (n = 61)[a] | Outside ESAs (n = 428) | Total (n = 489) |
|---|---|---|---|
| Before conversion | 26 ± 10 | 23 ± 6 | 23 ± 5 |
| After conversion | 12 ± 4 | [b] | 21 ± 5 |

[a] n = number of farms.
[b] The farms outside ESAs were not converted and have the same energy uses as before.

**TABLE 5**
**Average Energy Use ($10^6$ J ha$^{-1}$ year$^{-1}$) for Conventional Crop Production[13]**

| | Grass/Clover | Cereals | Row Crops | Perm. Grass |
|---|---|---|---|---|
| Oil[a] | 3.1 | 4.5 | 13.2 | 0.8 |
| Electricity[b] | 0.8 | 0.9 | 0.4 | 0.0 |
| Fertilisers[c] | 10.3 | 5.9 | 4.3 | 0.7 |
| Machinery | 1.0 | 1.4 | 4.0 | 0.3 |
| Total | 15.2 | 12.7 | 21.9 | 1.8 |

[a] Diesel, petrol, lubricants, etc. incl. refining and distribution.
[b] Irrigation and drying.
[c] Fertilisers, pesticides, and lime.

**TABLE 6**
**Average Energy Use ($10^6$ J ha$^{-1}$ year$^{-1}$) for Organic Crop Production[13]**

| | Grass/Clover | Cereals | Row Crops | Perm. Grass |
|---|---|---|---|---|
| Oil[a] | 2.4 | 4.3 | 11.3 | 0.8 |
| Electricity[b] | 0.8 | 0.7 | 0.5 | 0.0 |
| Fertilisers[c] | 0.0 | 0.0 | 0.0 | 0.0 |
| Machinery | 0.7 | 1.3 | 3.4 | 0.3 |
| Total | 4.0 | 6.3 | 15.2 | 1.1 |

[a] Diesel, gasoline, lubricants, etc. incl. refining and distribution.
[b] Irrigation and drying.
[c] Fertilisers, pesticides, and lime.

**TABLE 7**
**Livestock Density (LSU ha$^{-1}$) at Farms with Cattle, Sheep, and Other Ruminants Compared to Farms with Pigs, Poultry, and Other Nonruminants in the Present Situation (P) and in the Scenario (S) with Conversion to Organic Farming in Environmentally Sensitive Areas (ESAs) with Special Interests in Clean Groundwater**

| | Within ESAs | | Outside ESAs | Total | |
|---|---|---|---|---|---|
| | P | S | P = S | P | S |
| Ruminants | 0.6 | 0.4 | 0.5 | 0.5 | 0.5 |
| Nonruminants | 0.9 | 0.8 | 0.5 | 0.6 | 0.6 |
| Total | 1.5 | 1.2 | 1.0 | 1.1 | 1.0 |

## RESULTS

The agricultural land use was dominated by cereals, but conversion to organic farming led to more areas with grass/clover and smaller areas with cereals and row crops (Table 2). This resulted in lower E-uses for crop production (Tables 5 and 6), primarily because of lower energy inputs for synthetic fertilisers, where grass/clover benefit from its ability to fixate N from the atmosphere. Also, grazing in the grass/clover fields required less energy inputs per produced output than did harvest of roughage

**TABLE 8**
**Average Simulated Energy Use (MJ LSU$^{-1}$) for Conventional (C) and Organic (O) Livestock Production**

| | Ruminants (Cattle, Sheep, etc.) | | Nonruminants (Pigs, Poultry, etc.) | |
|---|---|---|---|---|
| | C | O | C | O |
| Electricity in livestock houses | 5.8 | 5.7 | 3.4 | 2.0 |
| Oil for heating livestock houses | 0.0 | 0.0 | 1.8 | 0.0 |
| Buildings, inventory, etc. | 2.7 | 2.7 | 2.7 | 2.7 |
| Fodder import | 5.4 | 4.2 | 12.9 | 9.7 |
| Own fodder production | 0.0[a] | 0.0[a] | 0.0[a] | 0.0[a] |
| Total | 13.9 | 12.6 | 20.8 | 14.5 |

[a] Included in the energy use for crop production.

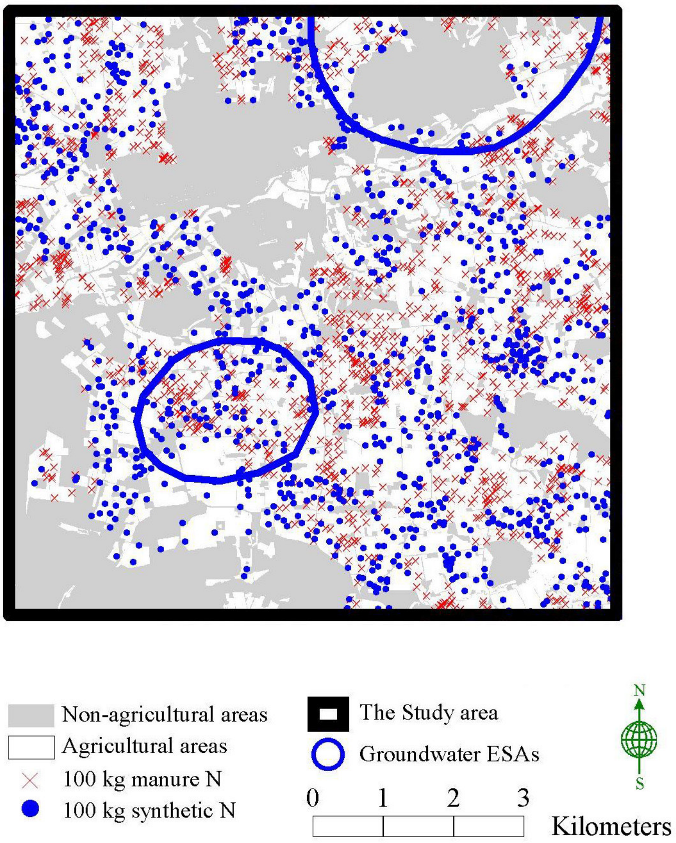

**FIGURE 4**. The geographical distribution of manure and synthetic fertilisers in the study area. Simulated average for a 30-year crop rotation with conventional farming.

fodder from whole-crop cereals and row-crop fodder beets. This contributed to lower E-uses in the organic than in the conventional crop rotations.

The animal production was reduced significantly with conversion to organic farming in the area with special interests in clean groundwater (Table 7). Thereby, the E-use for animal production was reduced via a combination of (1) reduced livestock density and (2) lower E-use per unit organic livestock compared to conventional livestock produced (Table 8).

Also, the manure production and the geographical distribution of fertilisers were affected by conversion. In the conventional situation, the field crops were fertilised with both animal manure and synthetic fertilisers (Fig. 4). In the organic situation, use of synthetic fertiliser was banned and the livestock production reduced. Thereby, the estimated N-surplus was also reduced (Table 2).

A statistical analysis of the simulated farm results revealed an average reduction of 10% in the N-surplus and a reduction of 54% in the E-use following conversion to 100% organic farming within the ESAs. A closer look at the confidence intervals revealed that none of these reductions were statistically significant at the 95% level, but the E-uses were very close to being significant (the confidence interval before conversion, 26 ± 10, nearly did not overlap that after conversion, 12 ± 4). Neither were the recorded average reductions in the N-surpluses or the E-uses in the total area significant, accounted to 1 and 9%, respectively (Tables 2 and 4).

## DISCUSSION

The simulated E-uses and N-surpluses in conventional compared to organic farming corresponded to empirical values from the literature. For example, Pimentel et al.[14] found an E-use of 10.0 and 7.2 GJ $ha^{-1}$, respectively, for conventionally and organically grown spring wheat in North Dakota. For Danish conditions, Vester[15] calculated the E-use of spring barley on organic model farms (6.9 to 13.0 GJ $ha^{-1}$) and on conventional farms (15.4 to 21.2 GJ $ha^{-1}$), while Leach[16] calculated typical E-uses in the U.K. and found an E-use of 15.7 GJ $ha^{-1}$ for spring barley, 26.4 GJ $ha^{-1}$ for the row crop maize, and 15.6 to 18.9 GJ $ha^{-1}$ for wheat. For animal production, Cederberg[17] found the same for dairy production in Sweden: 22.0 GJ $LSU^{-1}$ conventional cow ex farm, and 17.1 GJ $LSU^{-1}$ organic cow ex farm. Also, Halberg[18], in a study of 15 Danish dairy farms over a 3-year period, found a significantly lower E-use for organically compared to conventionally produced milk. Dalgaard et al.[3] and Halberg[18], respectively, reported N-surpluses of 124 and 114 kg N $ha^{-1}$ $year^{-1}$ for organic and 240 and 176 kg N $ha^{-1}$ $year^{-1}$ for conventional Danish dairy farms. This difference is larger than that presently estimated within the ESAs (Table 2). However, this can be explained by smaller differences in livestock density before and after conversion in the present study (Table 7) than in the two studies from literature, where organic farming with 1.1 LSU $ha^{-1}$ was compared to conventional farming with 1.7 LSU $ha^{-1}$.

The presented changes in N-surplus and E-use should be compared to the corresponding changes in production. The animal production changes are indirectly indicated with the accounted livestock density before and after conversion (Table 7). The arable crop production was not accounted specifically but corresponded to a 12 to 33% reduction in the crop yields following conversion to organic farming[10]. In the comparison of different scenarios for conversion to organic farming, or scenarios for other agricultural production changes[19,20], these costs in the form of reduced production should consequently be evaluated with the environmental benefits in the form of reduced N-losses and E-uses. However, the case is not straightforward — for example, 1 l of organic milk might be worth more than 1 l of conventionally produced milk, and the question of weighting quality appears. Such weighting is a subject for future research.

In conclusion, the method to map, simulate, and compare regional scenarios for N-surplus and E-use in conventional and organic farming was consistent under Danish and similar conditions. The results from a pilot scenario in the 10- ´ 10-$km^2$ study area indicated that conversion to organic farming in the environmentally sensitive areas with special interests in clean groundwater would reduce both N-surplus and E-uses. However, these reductions following the predicted changes in crop rotations, livestock densities, and fertilisation practices were not large enough to ensure a statistically significant reduction, though the energy use reductions nearly were significant at the 95% level. We therefore recommend further research in how conversion to organic farming, or other changes in the agricultural practice, might help to reduce N-surpluses and E-uses. In that context, the presented scenario tool in association with geographical information systems would be useful.

## ACKNOWLEDGEMENTS

Thank you to the Danish Ministry of Food, Agriculture, and Fisheries and The Danish Ministry of Environment and Energy for funding the research project Land Use and Landscape Development Illustrated with Scenarios (ARLAS), which made this publication possible.

| | | |
|---|---|---|
| Received: | July | 12, 2001 |
| Revised: | October | 8, 2001 |
| Accepted: | October | 15, 2001 |
| Published: | November | 16, 2001 |

## BIOSKETCH

**Tommy Dalgaard** is a Scientist with the Danish Institute of Agricultural Sciences in Denmark. In 1996 he received his M.Sc. in agroecology, and in 2001 completed his Ph.D. thesis, Simulation and Generalisation of Agricultural Resource Use. His work focuses on agricultural systems analyses. The aim is to integrate data in Geographical Information Systems, and to use these detailed data for bottom-up simulation and mapping of indictors for agricultural production. Also interactions with environment, economy, nature and society are included in the analysis. He has been an invited speaker at the International workshop, "Dissipation of N from the Human N-Cycle, and its Role in Present and Future $N_2O$ Emissions", Oslo (co-sponsored by the Intergovernmental Panel on Climate Change, IPCC), and at the EU-statistics seminar, "How Can Agricultural Statistics Meet Environmental Information Needs", Copenhagen. Dr. Dalgaard has been an invited expert for The Danish Parliament's committee for Nature and Environment, "Hearing on Loss of Nutrients to the Aquatic Environment", and "2nd European Invitational Expert Seminar on Life Cycle Assessments of Food Products", The Hague, The Netherlands.
